# Pediatric Formulations Developed by Extrusion-Based 3D Printing: From Past Discoveries to Future Prospects

**DOI:** 10.3390/pharmaceutics16040441

**Published:** 2024-03-22

**Authors:** Veronica Ianno, Sarah Vurpillot, Sylvain Prillieux, Philippe Espeau

**Affiliations:** 1 CNRS, INSERM, Chemical and Biological Technologies for Health Group (UTCBS), Université Paris Cité, 75006 Paris, France; philippe.espeau@u-paris.fr; 2 Delpharm Reims, 51100 Reims, France; sarah.vurpillot@delpharm.com (S.V.); sylvain.prillieux@delpharm.com (S.P.)

**Keywords:** pediatric formulation, children, 3D printing, oral dosage forms, personalized medicine, extrusion-based technologies

## Abstract

Three-dimensional printing (3DP) technology in pharmaceutical areas is leading to a significant change in controlled drug delivery and pharmaceutical product development. Pharmaceutical industries and academics are becoming increasingly interested in this innovative technology due to its inherent inexpensiveness and rapid prototyping. The 3DP process could be established in the pharmaceutical industry to replace conventional large-scale manufacturing processes, particularly useful for personalizing pediatric drugs. For instance, shape, size, dosage, drug release and multi-drug combinations can be tailored according to the patient’s needs. Pediatric drug development has a significant global impact due to the growing needs for accessible age-appropriate pediatric medicines and for acceptable drug products to ensure adherence to the prescribed treatment. Three-dimensional printing offers several significant advantages for clinical pharmaceutical drug development, such as the ability to personalize medicines, speed up drug manufacturing timelines and provide on-demand drugs in hospitals and pharmacies. The aim of this article is to highlight the benefits of extrusion-based 3D printing technology. The future potential of 3DP in pharmaceuticals has been widely shown in the last few years. This article summarizes the discoveries about pediatric pharmaceutical formulations which have been developed with extrusion-based technologies.

## 1. Introduction

Everything began in the 1980s with the invention and the development of the first 3D printing technologies such as selective laser sintering (SLS) and fused deposition modeling (FDM) [1,2,3]. More specifically, in 1989, fused deposition modeling (FDM) was originally invented and patented by Stratasys co-founder Scott Crump [4]. The rapid expansion of 3D printing technology has given a new edge to introduce innovative medical devices and customized drug delivery systems.

Most drug products are typically manufactured in large quantities using conventional methods that involve large-scale processes and equipment and long production times. The pharmaceutical industry is interested in using this 3D printing technology to overcome conventional mass manufacturing in order to develop personalized pharmaceutical therapy [5].

Three-dimensional printing is a form of additive manufacturing in which a three-dimensional object is built by depositing building materials in successive layers according to a predesigned three-dimensional geometric structure. Three-dimensional printing of pharmaceuticals is a unique approach that allows for the manufacture of solid drug products in various shapes, geometric designs, strengths and spatial distributions of the active pharmaceutical ingredients. Three-dimensional structures ranging from simple one-compartmental designs to complex multi-compartmental designs can be produced. The release profile of the active ingredients from these complex 3D drug products can be tailored to meet the needs of specific patients [6].

Moreover, three-dimensional printing (3DP) technology allows the adjustment of release kinetics and of individual doses, providing significant benefits for pediatric patients. Individual release characteristics may be required in this patient group in addition to dose adjustment. For instance, children may need special or smaller doses beyond what is conventionally available, or they may need unique dosage forms other than the standard pill, which can be difficult to swallow. Older adults may have various physiological or metabolic conditions due to certain illnesses or resulting from taking multiple medications, which may require alterations in doses or dosage forms on an ongoing basis. Moreover, it may be possible to combine certain medications into one “polypill” using 3D designs and 3D printing processes, which would be especially useful for those who take multiple medications every day [7,8].

All of these factors have also increased interest in 3DP technology in the pharmaceutical industry for its wide range of applications and versatility. Furthermore, the commercial feasibility of the Spritam^®^ 3DP tablet, an antiepileptic drug developed by Aprecia Pharmaceuticals (Blue Ash, OH, USA) and approved by the FDA in August 2015, indicates the potential for the commercial feasibility of manufacturing fast-dissolving oral tablets with high drug-loading profiles using ZipDose additive manufacturing technology. It is dispersed in the mouth with a very small amount of water in less than 10 s, making it very easy to use in populations of disadvantaged patients (e.g., pediatric patients or elderly patients) [9].

On the other hand, 3DP comes with the burden of challenges such as the requirements for excipients, the development of printing software and instrumentation, optimizing the mechanical properties of products and the regulatory landscape [10].

Unlike conventional methods of manufacturing that are required for so-called “blockbuster drugs” and create billions of tablets per year for global supply, 3D printing is already, or will soon be, well suited to supply orphan or smaller oncology indications, where only millions of tablets are required per year.

One of the purposes of this review is to summarize the discoveries made in recent years using extrusion-based 3D printing techniques to develop pediatric pharmaceutical forms. The other is to highlight the potential of 3D printing in revolutionizing conventional manufacturing toward small batch manufacturing (flexible and tailored pharmaceutical forms) and its advantages, such as safer and more effective treatments.

## 2. Materials and Methods

Several approaches were used to review the literature on 3D-printed oral pharmaceutical formulations. Three main databases, PubMed, Google Scholar and SciFinder, were used for this research. The keywords used in the search were as follows: 3D printing, oral solid dosage forms, pediatrics, personalized medicine, extrusion-based technologies, GMP-3DP. Finally, the Food and Drug Administration (FDA) and European Medicines Agency (EMA) websites were used to obtain updated information on pediatric pharmaceutical manufacturing guidelines.

At the beginning, the insight report of the Chemical Abstracts Service (CAS) Content Collection^TM^ was consulted to obtain a comprehensive and broader overview of 3D printing in biomedicine. Journal and patent publications were reported, categorizing them by the different years, countries, 3D printing technologies and materials used. Regarding the journal publications, the top contributing countries were China and the United States. From the annual trends of journal publications, a significant increase was shown from 2018, intensifying in 2021 for 3D printing applications [11].

**Figure 1:**
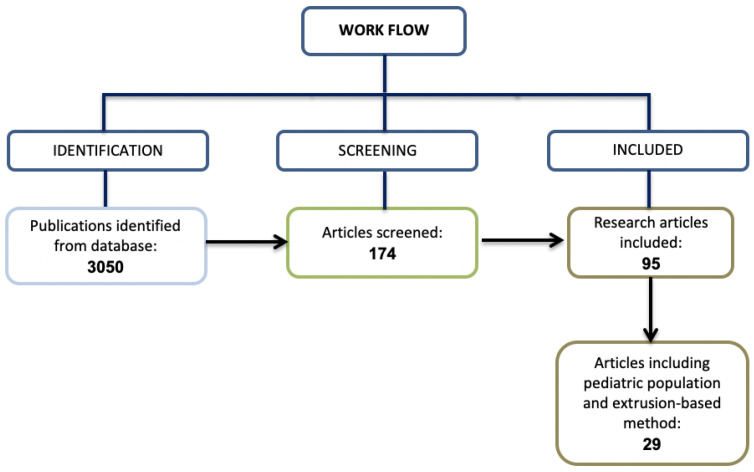
Literature selection.

Afterwards, from a total of 3050 results, only 174 were considered. These numbers were retrieved from the Scopus^®^ website using the term “3D printing and pediatric and extrusion-based” [12]. These publications had the following subject areas of interest: pharmacology, toxicology, pharmaceutics, material science, engineering and chemistry. The population regarded children of all ages. The articles (n = 27) which included a pediatric population and an extrusion-based 3D printing method were reported (Figure 1).

**Figure 2:**
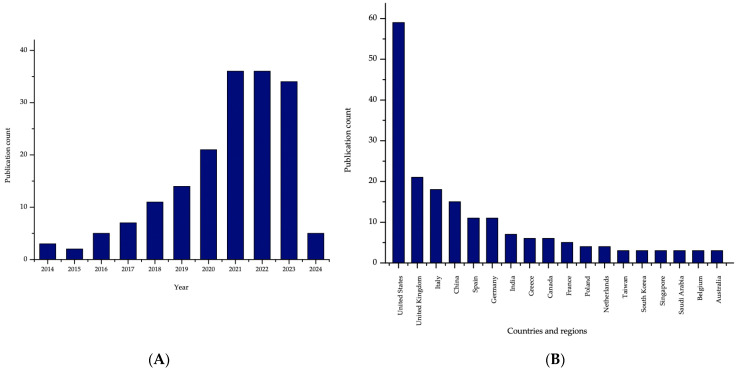
Number of journal publications relating to 3D printing in pediatric population (Scopus^®^) (**A**): over the last 10 years and (**B**): in the top 18 countries.

In addition, two graphs are shown to demonstrate the importance of increasing pediatric research through the number of journal publications around the world and in different years (Figure 2).

## 3. 3D Printing in Pharmaceutical Areas

**Figure 3:**
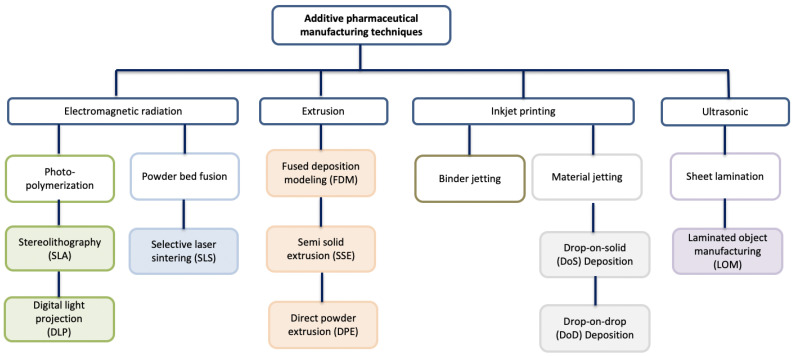
Overview of the classification of the 3D printing technologies used in pharmaceuticals.

Three-dimensional printing technology can be divided into four main principles, inkjet systems, nozzle-based deposition systems, electromagnetic systems and ultrasonic systems, which can be further divided into subtypes dependent on materials and methods (Figure 3) [13,14]. In the following sections, the most prevalent 3DP technologies for pharmaceutical applications are detailed. In addition, an overview of current progress in 3D printer systems and various pharmaceutical formulations is provided (Table 1).

### 3.1. Comparison of the Representative 3DP Technologies in Pharmaceutical Applications

**Figure 4:**
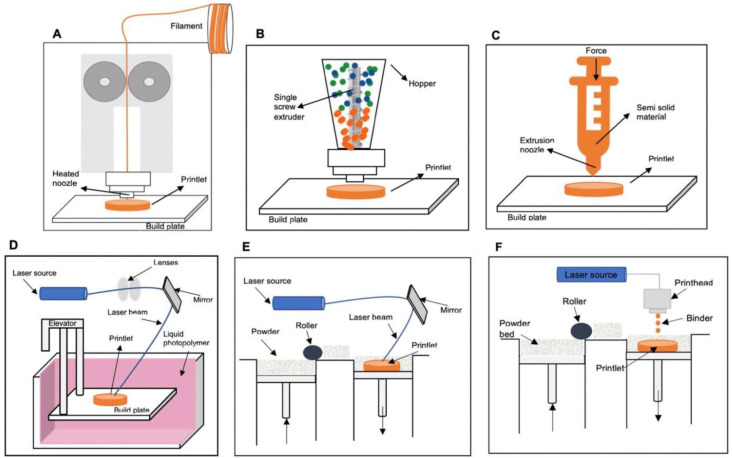
Schema of 3D printing technologies. (**A**): FDM 3D printing; (**B**): DPE 3D printing; (**C**): SSE 3D printing; (**D**): SLA 3D printing; (**E**): SLS 3D printing; (**F**): Binder jetting 3D printing.

Several 3D printing technologies have been developed with different characteristics. The 3DP technologies can be categorized into six groups: photo-polymerization-based printing, powder-based printing, extrusion-based printing, binder jet printing, material jet printing and lamination-based printing [15,16]. The most widely used 3DP technologies to develop pharmaceutical oral solid dosage forms include fused deposition modeling (FDM), semi-solid extrusion (SSE), direct powder extrusion (DPE), selective laser sintering (SLS) and stereolithography (SLA) (Figure 4).

#### 3.1.1. Fused Deposition Modeling (FDM)

FDM technology is based on a drug-loaded filament which is extruded through a heated nozzle. The printer head is moved along the x-y axis to release the fused extrudate, which solidifies at room temperature into a build plate [5]. In the literature, many advantages of coupling HME with FDM 3DP are shown when used in pharmaceutical applications. For example, they can improve the solubility of poorly soluble drugs (by producing amorphous solid dispersions) and their capability of manufacturing immediate- and sustained-release formulations [17]. Another advantage is to have a multi nozzle so that multi-combination drugs can be produced. On the other hand, the disadvantages of this technique include its unsuitability for thermosensitive drugs, the initial formulation of the filament and low drug loading. This printing technique is very cheap [18,19].

#### 3.1.2. Direct Powder Extrusion Technology (DPE)

Direct powder extrusion technology (DPE) overcomes the pitfalls of existing 3DP technologies. In this approach, a drug-loaded formulation blend is inserted into a powder hopper which can be processed with a direct powder extruder nozzle for the printing of the designed dosage forms without the preparation of drug-loaded filaments [20]. The hopper feeds into a heated single-screw extruder in the print head, producing a fused extrudate. Its advantages are the ability to manufacture immediate- and sustained-release formulations and to improve the solubility of poorly soluble drugs. A drawback of this approach is its unsuitability for thermosensitive drugs.

#### 3.1.3. Semi-Solid Extrusion (SSE)

The starting material is a semi-solid material (e.g., gel or paste). A drug-loaded semi-solid material is extruded through a heated syringe-based tool. The extrusion technique is based on the deposition of a gel or paste in sequential layers to create the 3D object. In particular, it is suitable for the production of chewable and palatable formulations [21]. An advantage of this method is its production of various formulation types with different forms of release, such as immediate and controlled or oral films and polypills. Unfortunately, this technique has low resolution and throughput. A further unfavorable point is the adaptability for drugs that can be formulated as semi-solids.

#### 3.1.4. Inkjet Printing

The inkjet printing requires materials with low viscosity, which makes it less applicable in manufacturing of oral solid dosage forms containing pharmaceutical-grade polymers [22]. The binder jet printing has a nozzle containing a binder liquid that moves along an x-y axis, depositing the liquid onto a flat powder surface. The liquid binds the powder particles together, leading to layer solidification [23,24]. This process is an expensive process and lacks portable equipment. Binder jet printing was used to develop the word’s first US Food and Drug Administration (FDA)-approved 3D-printed tablet.

#### 3.1.5. Stereolithography (SLA)

This process involves the exposure of a photo-polymerizable resin to high-energy light, such as UV light, to induce the polymerization and solidification of the material. SLA technology requires the development of drug-loaded photocurable resins, which also limits the choices of pharmaceutical-grade polymers that can be used. Huge disadvantages are potential issues around material toxicity and also the method’s unsuitability for thermosensitive and photosensitive drugs. Conversely, this technique is widely used to produce sustained-release drug formulations and medical devices due to its high resolution, which can be used to manufacture complex geometries [25,26].

#### 3.1.6. Selective Laser Sintering (SLS)

This process uses a laser that is directed to draw a specific pattern on the powder bed, leading to the selective partial or full melting of the powder particles. Once the layer is created, a roller distributes a fresh layer of powder on top of the created material. This process is repeated layer by layer to produce a 3D-printed medicine [27].

SLS technology may be unsuitable for photosensitive and thermosensitive drugs and requires precise control over powder flow characteristics. Another disadvantage is the requirement for post-processing. However, SLS has a great ability to produce highly porous dosage forms that dissolve quickly and different release formulations with a high-resolution process [16].

To conclude, extrusion-based technologies like FDM, SSE and DPE are probably more suitable for printing at dispensing points in hospitals and pharmacies. The other technologies could be more suitable for printing drug-loaded medical devices. The combination of HME and FDM technologies could be particularly useful for manufacturing customized solid oral formulations quickly, reliably and inexpensively [28]. Due to these economic and rapid prototyping aspects, 3D printing has attracted a lot of interest from the pharmaceutical research field.

### 3.2. Availability of Pharmaceutical Materials

The choice of the materials used in formulation development is equally important as the selection of the technology. Especially in pediatric formulations, this choice is a key factor for safety and dose acceptability, as infants and children are physiologically different from adults [6].

There are now a wide variety of different material types which are supplied in different states, such as powder, filament, pellets, granules, resin, etc. Nowadays, various pharmaceutical polymers have been commercialized, including for pediatric use, such as cellulose ethers (hydroxypropyl cellulose (HPC); hydroxypropylmethylcellulose (HPMC); ethylcellulose (EC); hydroxypropyl-methylcellulose acetate succinate (HPMCAS)), derivates of acrylates (e.g., Eudragit^®^) and polyvinylpirrolidone (PVP). In Yochana et al.’s review, an overview of the classifications of pharmaceutical excipients used in the pediatric population and their possible substitutions is outlined, highlighting their safety and toxicity [29,30]. On the other hand, in Pereira et al.’s review, a detailed list of pharmaceutical polymers traditionally used in the HME and FDM techniques is provided (e.g., polylactic acid (PLA); acrylates; polyvinyl alcohol (PVA), etc.) [31].

Moreover, the challenges in material selection are drug compatibility, biodegradability and acceptability for 3DP formulation manufacture. In addition, the material must not produce any toxic substances during or after the manufacturing process. Although many different materials and methods are available, the lack of novel and innovative materials leads to slow advancement in various pharmaceutical applications.

### 3.3. Upcoming 3DP Systems for Personalized Medicine

Most current 3D printing methods and instruments are not specifically designed and developed for pharmaceutical applications. There are several disadvantages that need to be overcome before the extensive application of 3D printing technologies can be realized in pharmaceutical areas. There is a need to design and develop 3D printing methods and equipment specifically for pharmaceutical applications. The needs in pharmaceutical applications for 3D printers include the precision and accuracy of each printed unit, reproducibility, use of pharmaceutic excipients without additional processing, coordination of printing multiple materials, high throughput and Good Manufacturing Practice (GMP) compliance. Currently, Aprecia, Triastek, FabRx and DiHeSys Digital Health Systems GmbH have developed GMP-compliant 3D printers specifically for pharmaceutical applications [32].

#### 3.3.1. FabRx Ltd.

In 2020, FabRx Ltd. (London, UK), created by University College London researchers to develop pharmaceutical 3D printing, launched the M3DIMAKER GMP printer. The printer was developed to design and manufacture personalized drugs in hospitals and pharmacies or to produce small batches for clinical trials. It is in use in several clinical trials around the world, including one at the Gustave Roussy Hospital in France, a cancer research center [33].

The M3DIMaker printer is a material extrusion type of 3D printer that melts a material and deposits it in layers on the printing bed to build up the desired structure. The printer has three different types of printheads (FDM, DPE, SSE) that can be used, which are developed in close communication with regulatory agencies, including the MHRA (Medicines & Healthcare products Regulatory Agency) and hospital users, depending on the material. Hence, one of the three heads can be used for fused deposition modelling (FDM) technology where the API and other ingredients are combined in an extruder and formed into a filament in a separate manufacturing step. Then, the filament is melted and deposited in the FDM printer. The second head allows printing with direct powder extrusion (DPE) technology, in which the ingredients in powder form are fed directly into the hopper of the 3D printer. Finally, the third head allows printing using the semi-solid extrusion (SSE) method, which extrudes a gel or paste from a syringe under pressure. SSE operates at a lower temperature than FDM or DPE, which makes it suitable for thermosensitive drugs [34].

The GMP features of the M3DIMAKER include quality control procedures and camera monitoring of the process, assuring that each 3D-printed tablet contains the dose that it should have.

#### 3.3.2. Triastek Inc.

Triastek (Nanjing, China) has recently launched a scaled-up melt extrusion system that enables the manufacturing of personalized drug products for clinical trials and for mass production. Triastek’s melt extrusion deposition (MED^®^) 3D printing technology mixes and deposits materials to produce solid drugs [35]. Triastek can control the placement, timing and speed of the deposition of drug components. Its 3D formulation by design (3DFbD^®^) patented method enhances the efficiency and success rate of its drug development and reduces side effects by altering and combining the release of various components of the printed drug [36]. Triastek Inc. announced a partnership with Boehringer Ingelheim China to deploy its MED^®^ technology to create multiple prototypes of the new chemical entity (NCE) using its 3D printing Formulation by Design (3DFbD^®^).

Other companies, including DiHeSys (Schwäbisch Gmünd, Germany), Vitae Industries (Providence, RI, USA) and Craft Health (Tulsa, OK, USA), are also working towards producing GMP-compliant pharmaceutical printers, but no research has yet been published using these printing systems. However, DiHeSys (Schwäbisch Gmünd, Germany) with Harro Höfliger are developing the FlexDosePrinter suitable for manufacturing 2D- and 3D-printed medicines, particularly Oral-Dispersible Films [37].

Both of these printer models (FabrX (London, UK), Triastek (Nanjing, China), Aprecia (Blue Ash, OH, USA), DiHeSys (Schwäbisch Gmünd, Germany)) use a higher-performance nozzle array design in contrast to a single one to overcome the issue of daily printing speed. For example, Triastek reported the ability to produce 150–200,000 tablets in a day [32].

### 3.4. State of the Art of the Clinical Development of 3D-Printed Drugs

The world’s first-in-human clinical study was completed by FabRx Ldt. (London, UK) and investigated at the Clinic Hospital at University De Santiago de Compostela, Spain. Chewable isoleucine printlets with six different flavors and colors and four different dosages were prepared using SSE technology to personalize medicine for children aged 3–16 years with several rare metabolic disorders, including maple syrup urine disease (MSUD). After six months of treatment, the 3D-printed formulations demonstrated more desirable isoleucine blood levels and improved medicine acceptability compared to standard isoleucine therapy (target value 200–400 μM). This study shows the possibility of preparing chewable formulations with different doses of isoleucine (50, 100, 150 and 200 mg) and flavors [38].

Research is ongoing at Alder Hey Hospital in Liverpool for the production of personalized 3D-printed hydrocortisone preparations for pediatrics [39].

In June 2021, FabRx and Gustave Roussy, an oncology hospital in Paris, announced a collaboration focused on bringing multi-drug 3D-printed formulations into the clinic. The aim of this collaboration is to develop and print an anticancer therapy in the form of a single dose-personalized tablet for the treatment of early-stage breast cancer patients [40].

Orally disintegrating printlets with Braille and moon patterns on the surface of the dosage forms to enable visually impaired patients to identify medications were manufactured using SLS technology [41].

Furthermore, three of Triastek’s own 3D-printed drug products, T19 (for rheumatoid arthritis), T20 (for cardiovascular and clotting disorders) and T21 (for ulcerative colitis), received FDA approval under Investigational New Drug (IND) clearance from the FDA in January 2021, March 2022 and November 2022, respectively. The company has started pharmacokinetic study trials with a clinical contract research organization. In addition, T29 has received IND approval from the NMPA (National Medical Product Administration) in China in July 2022 [42].

Nevertheless, Spritam, made by Aprecia Pharmaceuticals (Blue Ash, OH, USA) using the ZipDose technology that received FDA approval in 2015, remains the only commercially available 3D-printed drug in the US.

**Table 1:** Summary of the types of pharmaceutical 3D printing technologies and their respective innovations.

3D Printing Systems		GMP-Manufactured Pharmaceutical Printer	FDA IND Clearance 3DP Drug	FDA Approval and Commercially Available 3DP Drug	Ref.
Extrusion-based					
	Fused deposition modeling	FabRx Ltd. (London, UK) °			
		DiHeSys (Schwäbisch Gmünd, Germany) °			
	Semi-solid extrusion	FabRx Ltd. (London, UK) °	Isoleucine chewable tablets, 2019		[38]
		Craft Health (Tulsa, USA) °			
	Direct extrusion printing	FabRx Ltd. (London, UK) °			
	Melt extrusion deposition (MED)	Triastek Inc. (Nanjing, China) *	T19, 2021 T20, 2022 T21, 2022		[42]
Powder bed fusion					
	Binder jetting	Aprecia Pharmaceuticals (Blue Ash, USA) *		Spritam^®^, 2015	[43]
	Selective laser sintering	FabRx Ltd. (London, UK) °			
Vat polymerization					
	Stereolithography	FabRx Ltd. (London, UK) °			
	Direct light processing				
Inkjet printing		DiHeSys (Schwäbisch Gmünd, Germany) °			
	Drop-on-solid				
	Drop-on-drop				
Lamination-based					
	Laminated object manufacturing				

°: customized small batches; *: large-scale manufacturing.

## 4. Extrusion-Based Discoveries in Pediatric 3D Printing Formulations

This section will highlight studies on the manufacturing of pediatric formulations using extrusion-based 3D printing technology, as shown in Table 2. This Table was divided into subgroups based on manufacturing techniques (SSE, DPE, FDM and hot melt pneumatic extrusion (HMPE)), the dosage form, the active pharmaceutical ingredient, the main matrix-former polymer and other components (plasticizer, disintegrant, filler/lubricant, …), the extruder and printer model with the name of the manufacturer, the temperature used (°C) and lastly the target release profile. The possibility of manufacturing “patient-specific” drug products with different shapes, sizes and releases is shown in these studies [44].

### 4.1. Palatable Personalized Pediatric Forms

Children’s difficulty swallowing oral solid dosage forms could be overcome by manufacturing medical gummies developed through 3D printing, due to their striking appearance and pleasant organoleptic characteristics [45].

In Scroutari et al.’s work, indomethacin Starmix^®^ “candy-like” chewable tablets were printed using FDM 3DP to improve patient compliance and palatability through various shapes such as lion, heart, teddy bear or circle. The purpose was to imitate Haribo sweets to prepare pediatric medicines that can be consumed easily by children from 2 to 11 years old [46].

However, the importance of the appearance, perceived taste and texture for children aged 4–11 years was demonstrated through a survey of visual preferences where the placebo tablets were printed using SLA, SLS, SSE and FDM methods [47]. Accordingly, Wang et al.’s research designed and fabricated donut-shaped tablets using FDM 3DP in pairs with HME technology in order to tailor taste masking and to improve compliance in the pediatric population [48]. Another study demonstrates that FDM can be used to produce innovative pediatric-friendly printed shapes such as red mini-waffle forms containing hydrocortisone [49].

On the other hand, it was demonstrated how starch-based soft formulations for the treatment of pediatric tuberculosis with isoniazid can be economically printed using SSE 3D printing in different shapes (blue cube, heart, moon and dog) [50].

The adaptability of 3D printing technology can be used for multi-drug formulations. An example is the Lego^TM^ bricks made of paracetamol and ibuprofen chewable soft material that were manufactured using SSE technology [51]. In addition, it is possible to print pharmaceutical formulations based on chocolate in different shapes resembling cartoon characters for pediatric applications [52].

More recently, cereal-based 3D-printed dosage forms have been suggested for pediatric use to overcome adherence barriers and to improve adherence to treatment in pediatric patients in a hospital setting. The cereals were crushed and mixed with the two active ingredients, paracetamol and ibuprofen, and after that, oral formulations with different shapes (e.g., various letters, star, heart, torus and flower shapes) were printed using the SSE technique. The creativity lies in hiding the drugs inside a common breakfast ingredient [53].

Therefore, in Herrada et al.’s study, different shapes (heart, teddy bear) were made more appealing in order to achieve a more final eye-catching form through color, bright smooth finished surfaces, gumminess and manipulability [54].

In conclusion, these studies have shown an alternative route of manufacturing palatable personalized pediatric forms. The strength of the 3D printer resides in its ability to create different shapes palatable to children.

### 4.2. Pediatric Formulation Release and Size

Several studies have focused on manufacturing child-size-acceptable formulations, including the relationship between the size and the release of the active ingredient. The 3D printing of minitablets enables dose individualization and variations in release behavior according to the size and polymer used.

For example, in a study by Kraus et al., minitablets containing caffeine and propranolol hydrochloride were printed and characterized using HydroxyPropylMethylCellulose (HPMC) or HydroxyPropylCellulose (HPC), PEG 6000 and fumed silica [55], whereas in 2019, Palekar et al. developed a modified-release bacoflen minicaplet, a spasmolytic drug, using PVA (polyvinyl alcohol) as the polymer with sorbitol [56].

Minitablets have been proven to be a safe, reliable and easy dose approach to administering oral medication to young children [57].

Through DPE technology, a minitablet containing approximately 1 mg of budesonide was produced for the treatment of eosinophilic colitis in pediatric patients [58].

A novel SSE printing technique has demonstrated to be able to precisely print furosemide and sildenafil tablets in smaller batch sizes [21].

### 4.3. Taste Masking

The adequate choice of taste-masking agents (i.e., sweeteners and/or flavoring agents) is essential to improve the palatability of oral dosage forms and patient compliance. Several studies have focused on this aspect. For example, in Tabriz et al.’s study, excellent taste masking of the bitter taste of chewable ibuprofen tablets was demonstrated, and in Khalid et al.’s work, hot melt printing demonstrated the ability to print palatable orodispersible films loaded with a thermosensitive bitter drug such as diclofenac [59].

Positive results of DPE 3D printing have been obtained in taste masking and possible dose reduction when customizing praziquantel medicines for pediatric patients. Also, the SSE technique was used to produce propranolol hydrochloride gummy chewable tablets to treat cardiac arrhythmias [60].

Additional fruit-flavored 3DP Diphenhydramine Hydrochloride (DPH) tablets were prepared in various shapes in order to mask the bitter taste of the active ingredient [61].

### 4.4. Challenge of Poorly Water-Soluble Drugs 

The challenge of oral forms is due to low solubility being one of the necessary factors required in absorption after a drug’s oral administration. Poorly water-soluble drugs often require high doses in order to reach therapeutic plasma concentrations after oral administration. According to the Classification of Biopharmaceutics (BCS), Class II and IV drugs exhibit poor solubility, lower bioavailability and less dissolution. One of the useful pharmaceutical techniques used to enhance solubility is solid dispersion by HME [62].

The solubilizer type and quantity in the formulation are influential in drug dissolution, and when the dose increases, dissolution also increases. For example, in Saydam et al.’s research in 2020, the increased dissolution of rufinamide was achieved through solid dispersion with solubility enhancers like Gelucire^®^ (44/14 and 48/16) and coupled HME and FDM 3DP technology. Rufinamide is an orphan drug indicated for the treatment of Lennox–Gastaut Syndrome (LGS), a rare form of childhood epilepsy [63].

In addition, Lumefantrine was used as a model poorly water-soluble active pharmaceutical ingredient and the Eudragit EPO was chosen as a matrix former with xylitol as a hydrophilic plasticizer and maltodextrin to achieve rapid dissolution. A rapid release criterion was set to 30 min at pH = 2 to dissolve at least 85% of the drug [64].

### 4.5. HME and FDM Coupling Potential

The feasibility and benefits of FDM 3D printing coupled with hot melt extrusion (HME) are shown in several studies as a new approach for manufacturing potential drug delivery systems for pediatric populations [28,48]. In addition, HME can provide taste masking of bitter active pharmaceutical ingredients (APIs) [46].

In 2021, Roulon et al.’s work demonstrated the possibility of using the HME and FDM methods at low temperatures: the extrusion temperature (from 50 °C to 80 °C) and the printing temperature (at 80 °C). The results of the research demonstrated that it was possible to produce fast-disintegration oral forms using amiodarone hydrochloride, an insoluble antiarrhythmic drug; polyethylene oxide (PEO) as a water-soluble thermoplastic polymer; D-sorbitol as a filler to produce fast disintegration; glycerol as a liquid plasticizer; and silicon dioxide (SiO_2_) as a gliding agent [65].

Although there are many advantages to using this coupling, in Malebari et al.’s work, ritonavir and lopinavir minispherical tablets were printed using a DPE 3D printer in order to show its efficacy in comparison with the HME and FDM methods. These two APIs were used in the initial first-line antiviral therapies in HIV-positive pediatric children. The formulation was prepared with HMPCAS, PEG 4000 and magnesium stearate. The high extrusion and printing temperatures and the drug degradation process at 120 °C were the reason why FDM was replaced with DPE. Using the DPE method, the temperature was decreased to 80 °C [66].

On the other hand, in Yang et al.’ s project, manufactured immediate-release hydrocortisone (HC) tablets allowed the use of personalized doses for children with congenital adrenal hyperplasia, HC having thermally labile activity and both extrusion and 3D printing temperature and drug concentration in the polymer satisfying the pharmacopeia limits for drug contents and impurity [28].

**Table 2:** Overview of the 3D-printed pediatric formulations based on extrusion-based technology.

Technique	Dosage Form	API	Polymer (s) and Other Excipients	T °C and Extruder Model	T °C and 3D Printer Model	Patient Acceptability	Target Release Profile	Ref.
FDM	Chewable tablets	Indomethacin BCS class II	PEG 6000, HPMCAS	40–120 °C Eurolab16, Thermo Fisher Scientific (Waltham, MA, USA)	165 °C HD2xR Airwolf (Costa Mesa, CA, USA)	“Candy-like” with various shapes and taste masking	Immediate release	[46]
	Minitablets	Baclofen BCS class III	PVA, sorbitol	160 °C Process 11, Thermo Scientific	170 °C Makerbot Industries (New York, NY, USA)	Size suitable for children	Sustained release	[56]
	Tablets	Rufinamide BCS class II	Kollidon^®^ VA64, HPMC, Soluplus^®^, triacetin, Gelucire^®^ (44/14 and 48/16)	120–150 °C Filabot EX2, Barre, VT, USA	215 °C CraftBot3, Budapest, Hungary	Improved dissolution	Enhanced drug release	[63]
	Tablets	Caffeine Citrate BCS class I	Eudragit^®^ EPO, HPC (LF and HF), HPMC K4M	155 °C Thermo Fischer	200 °C Prusa i3 3D printer (Prague, Czech Republic)	Taste-masked formulation	Modified release	[48]
	Tablets	Amiodarone BCS class II	D-sorbitol, PEO, glycerol, SiO_2_	50–80 °C Thermo Fischer	80 °C Prusa i3 Mk3S 3D printer	Reproducible filament batches for oral formulation	Immediate release	[65]
	Tablets	Lumefantrine BCS class II	Eudragit^®^ EPO, Xylisorb 300, maltodextrin	>100 °C Pharma11, Thermo Scientific	160 °C BoltPro 3D-printer, Alphen aan den Rijn, The Netherlands	Size suitable for children > 6 years	Immediate release	[63]
	Minitablets	Caffeine and propranolol hydrochloride BCS class II and I	HPMC, HPC, PEG 6000	HPMC 120 °C//HPC 90 °C Turbula T2F, System Schatz, Lauterhofen, Germany	HPMC 200 °C//HPC 170 °C Ultimaker, Zaltbommel, The Netherlands	Size suitable for children	Dependent on tablet size	[55]
	Chewable tablets	Diphenhydramine Hydrochloride BCS class I	Hydroxypropyl cellulose, Gelucire^®^ 48/16, sucralose, strawberry flavour	120 °C Eurolab16, Thermo Fisher	165 °C Ultimaker 3 extended, The Netherlands	Taste masking	Immediate release	[61]
	Tablets	Hydrocortisone BCS class I	Eudragit^®^ EPO, Triethyl citrate, talc, sodium stearyl fumarate, TiO_2_	110 °C Thermo Fisher	140 °C MarkerBot Industries (New York, NY, USA)	Size suitable for children and lower drug contents	Immediate release	[67]
	Tablets	Hydrocortisone BCS class I	Kollidon^®^ VA64, Affinsol^®^ 15LV, PEG 6000, Sorbitol, Triethyl citrate	90–140 °C Thermo Fisher	155–180–210 °C Prusa i3MK3 3D desktop printer (Czech Republic)	Pediatric-friendly printed shapes	Personalized sustained release	[49]
FDM and DPE	Minitablets	Ritonavir and Lopinavir BCS class IV	PEG 4000, HPMCAS, magnesium stearate	120 °C Noztek (Shoreham, UK)	80 °C DPE: Hyrel 3D SR (Norcross, GA, USA)	HIV-infected children	Zero-order sustained release profile over 24h	[66]
DPE	Tablets	Praziquantel BCS class II	Kollidon^®^ VA 64, Surfactants (Span™ 20 or Kolliphor^®^ SLS)	50–180 °C Thermo Fisher	140–200 °C physical mixtures; 130–140 °C pellets and milled powder obtained from HME FabRx Ltd., London, UK	Masking of taste	Increased API release	[20]
	Minitablets	Budesonide BCS class II	PEG6000, HPMC, HP-β-CD	-------	180 °C 3DForMe^®^ printer (Farmalabor, Italy)	Size and dose suitable	Sustained colonic release	[58]
	Orodisperdible films	Clobetasol propionate BCS class II	PEO 100.000, HPMC, HP-β-CD, chitosan	-------	170 °C 3DForMe^®^ Printer (Farmalabor, Italy)	Mucoadhesive films	Sustained release	[68]
HMPE	Chewable tablets	Ibuprofen BCS class II	Soluplus^®^, Kollidon ^®^ VA64, Eudragit^®^ EPO	-------	IBU/VA64: 120 °C, IBU/EPO: 90 °C IBU/Soluplus: 105 °C Bio-X (Celink, Sweden)	Taste masking	Specific release profile tailored to individual patient	[69]
SSE	Chewable printlets	Isoleucine BCS class II	Sucrose, pectin, maltodextrin, water, flavourings and colorants	-------	70 °C The magic candy factory	Patient acceptability and colour and flavour patient choice	Immediate release	[38]
	Chewable dosage form	Paracetamol and Ibuprofen BCS class I and class II	Glycerol, gelatin, locust bean gum, water, food dyes	-------	75 °C Makerbot Industries	Lego™-like brick shapes	Dual drug release	[51]
	Chewable dosage form	Paracetamol and Ibuprofen BCS class I and class II	Bitter chocolate, corn syrup	-------	45 °C 3D Food Printer (Model 3C10A)	Cartoon character shapes and palate acceptability	Immediate release for paracetamol and pH- dependent for Ibuprofen	[52]
	Chewable dosage form	Ranatidine BCS class III	Xanthan gum, strawberry essence, gelatin, corn starch, carrageenan, food colouring	-------	37 °C A syringe-based extrusion 3D printer (bIDO-I, Idonial Technological Centre, Gijón, Spain)	To improve the organoleptic characteristics and palatability of the formula	Immediate- and sustained-release profiles	[54]
	Tablets	Furosemide and sildenafil citrate BCS class IV and class II	Gelucire^®^ 48/16, Polysorbate 80	-------	40–41 °C A modified Prusa i3 MK2 3DP	Smaller batch size suitable for children	Immediate release	[21]
	Oro-dispersible film	Diclofenac BCS class II	Maltodextrin, glycerol, TiO_2_, mint and licorice flavors, sucralose	-------	95 °C A modify FDM 3DP (Futura Group Srl, Gallarate, Italy)	Taste-masking	Immediate release	[59]
	Tablets	Isoniazid BCS class III	Corn starch	-------	Cellink-ink- redible printer (Gothenburg, Sweden)	Pediatric-friendly formulations	Immediate release	[50]
	Chewable tablets	Propranolol hydrochloride BCS class I	Gelatin, carrageenan, sodium carboxymethyl starch	-------	80 °C FoodBot (Hangzhou, China)	Size and shape suitable	Immediate release	[60]
	Oro-dispersible tablets	Loratidine BCS class II	Cellulose, mannitol, carboxy methyl starch sodium, Kollidon^®^ 30	-------		Suitable for elders and children with dysphagia	Immediate release	[70]
	Oro-dispersible minitablet	Carbamazepine BCS class II	Ac-Di-Sol^®^, Kollidon^®^ 30, sucralose, lactose monohydrate	-------	Bioprinter Bio XTM (Cellink, Gothenburg, Sweden)	Taste masking	Sustained release	[71]
	Tablets	Caffeine BCS class I	Sodium alginate, HPMC, sodium croscarmellose	-------		Body-weight-adjusted dosage, neonate targets	Rapid release	[72]
	Chewable tablets	Amlodipine besylate BCS class I	Carboxymethyl cellulose sodium, sodium starch Glycolate, glycerin	-------		Taste masking and cartoon shape	Immediate release	[73]
	Printles	Hydrochlorothiazide BCS class III/IV	Sucrose, PVP, banana flavoring essence, croscarmellose sodium, lactose	-------	FabRx M3dimaker (London, UK)	Personalized medicines with high drug load	Oro- dispersible printlets	[74,75]
	Tablets	Levetiracetam	Kollicoat^®^ IR,	-------	27 °C 3D-Bioplotter (EnvisionTec, Gladbeck, Germany)	Dose suitable	Immediate release	[76]

Abbreviations: DPE: direct powder extrusion; FDM: fused deposition modeling; HME: hot melt extrusion; SSE: semi-solid extrusion; PVA: polyvinyl alcohol; PEG: polyethylene glycol; HPMCAS: hydroxypropylmethylcellulose acetate succinate; HPMC: hydroxypropyl methylcellulose; HPC: hydroxypropyl cellulose; PEO: polyethylene oxide; HP-β-CD: 2-Hydroxypropyl-β-cyclodextrin; BSC: Biopharmaceutical Classification System; PVP: polyvinylpyrrolidone; -------: extruder not used.

## 5. Benefits and Limitations of Various Aspects of 3D Printing Formulation

The consideration of children’s preferences regarding the shape and color of the dosage form, precise doses and personalized doses and dosage forms are some of the benefits of these technologies. Moreover, in the previously cited studies, the manufacturing of personalized polypills was shown to be possible by means of 3D printing, leading to a significant advantage in the treatment of several medical conditions and improved adherence to medications, such as in cases of polypharmacy [77].

An ideal immediate-release 3DP product for children would be as small as possible, would be easy to identify to prevent medication errors, could be stored at room temperature and would have adequate shelf life. The manufacturing method must enable production within a short time. Therefore, dose verification prior to drug administration and the medication safety of the drug product (e.g., the excipients, the colorant) are considered very important factors and prerequisites for the adoption of 3D printing in hospital settings.

### 5.1. Benefits of 3D Printing of Pediatric Formulations

Nowadays, many pediatric formulations are not available in suitable oral solid dosage forms. Changes in physical, metabolic and psychological processes that occur during children’s growth, from birth to adulthood, suggest that children should not be considered as young adults. Therefore, pediatric patients require different delivery systems, changing dosing and administration requirements [78]. The lack of adequate medicines developed for children results in several off-label and unlicensed prescriptions, increasing the risks of adverse drug reactions. Furthermore, commercially available fixed-dose combinations can result in under- or over-dosing of one component in different pediatric patients, and the cutting of tablets does not always result in accurate doses. Tablets are split manually, leading to imprecise dosage division and drug delivery problems such as the early release of the drug or the breakage of the tablet coating. In addition, the stability of some drugs at certain concentrations, the storage conditions and the appropriate use of excipients are difficult criteria to satisfy in extemporaneous liquid preparations [10]. For example, in Zheng et al.’s research, the potential to use 3D printing technology as a new method for hospital dispensing was demonstrated through a comparison of pharmacists’ split subdivided tablets with 3D-printed spironolactone tablets. The results showed that the 3D-printed spironolactone tablets were more accurate, safer and more personalized [79].

Dose adjustment continues to remain an important issue and a growing need. Three-dimensional printing technology has a great future application as a precise and safe dispensing method for subdivided drugs which could replace traditional technology, particularly useful in pediatric patients. In addition, 3D printers might be the solution to these issues when approaching personalized pharmacotherapy [4].

Pediatric oral formulations need to be improved. This is an indisputable fact that has gained attention from regulators, medical staff and researchers.

According to the EMA and Alessandrini et al.‘s survey, the conventional dosage forms, i.e., liquids (35%), tablets (19%) and capsules (14%) were the most frequently selected [80]. Specifically, before the age of 1 month, the oral form has to be in a liquid phase in order to be mixed with baby food or to be directly drunk, whereas between 1 month and 2 years old, orodispersible forms become acceptable, and after 2 years old, the use of tablets is a possibility. Three-dimensional printing technology is still suitable for the preparation of tablets for children aged 2 years old and over [81,82].

### 5.2. Regulatory Landscape

The implementation of 3DP for tailored medicines faces several challenges, notably for regulatory agencies. Despite the high interest in extrusion-based processes in the medical field, the availability of Good Manufacturing Practices (GMPs) for 3D printers, software and input materials as pharmaceutical filaments still remains very limited. Pharmacopoeias do not contain methods of analysis for tablets produced by 3DP and the existing methods for the other pharmaceutical oral solid dosage forms are difficult to apply [63]. Although the polymers used to date are generally recognized as safe in adults, the nature and doses tolerated in children may be different and must be ensured. Thus, pediatric tolerance studies and the development of new polymeric excipients will go hand in hand with the introduction of 3DP into pediatrics. To discover regulatory issues in the printing process of pharmaceutical formulations, a concrete regulatory requirement for printable products is urgently needed [55].

Although drug therapy plays an essential role in the management of infants and children suffering from a variety of acute or chronic diseases, the majority of drugs approved by the Food and Drug Administration (FDA) are for adults and have not been approved for use in pediatric patients. One of the major impediments to their use, however, is the lack of suitable pediatric oral solid dosage forms.

Moreover, the primary goal of the E.U. and U.S. legislation is identical: to improve children’s health through advancements in research and to provide a framework for the evaluation of efficacy and safety in the pediatric population. 

Nowadays, the FDA is investigating and regulating 3D-printed products in accordance with the current regulations. Good Manufacturing Practice (GMP) guidelines for 3D-printed pharmaceuticals have not been yet established, but in 2017 the FDA established guidelines for the 3D printing of medical device products [83].

Before the Pediatric Regulation from the European Medicines Agency came into effect in 2007, many medicines authorized in Europe were not studied adequately or authorized in children [84]. This caused difficulties for prescribers and pharmacists treating children, as well as for their patients and carers. The Paediatric Regulation came into force in the European Union (EU) on 26 January 2007. Its objective is to improve the health of children in Europe by facilitating the development and availability of medicines for children aged 0 to 17 years. The main impact of this regulation was to establish the Paediatric Committee (PDCO). Its main role was to provide objective scientific opinions on pediatric investigation plans (PIPs) and to develop plans for pediatric medicines [85].

An important pharmaceutical strategy for Europe was the reform of the EU pharmaceutical legislation. In April 2023, the commission adopted a proposal for a new Directive and a new Regulation. It revises and replaces the existing pharmaceutical legislation, including the legislation on rare diseases and children, which was finally disclosed [84].

Because of the complexity of clinical studies and the elaborate approval process, it will take time, but the European legislature has already changed a great deal concerning pediatric medicine. Overall, the various political incentives in both the European Union and the United States seem to be bringing changes in pediatric drug formulations [57,86].

### 5.3. Market Landscape

The global drug shortage associated with the COVID-19 pandemic has powered the fast growth of 3D printing in the pharmaceutical field. This growth is being fueled by the emergence of new industry players, as well as the release of new 3D printers, materials and start-up funding. Some of the major players operating in the market include Aprecia Pharmaceuticals, GlaxoSmithKline plc, AstraZeneca, FabRx Ltd., Hewlett Packard Caribe and Merck KGaA [87].

In 2021, a 3D-printed drug market overview was conducted to show a compound annual growth rate (CAGR) growth of 15.2% from 2021 to 2027, projected to finally reach USD 2064.8 million by 2027 compared with USD 638.6 million in 2019. It also proves how the tablets captured the largest market share and accounted for 29.8% of the market revenue. The tablets’ advantages are in longer shelf life, greater variety of shapes and higher doses of the active pharmaceutical ingredients [88].

Amongst the end-users, research laboratories dominated the global 3D-printed drugs market with nearly 51.7% of the market share [88].

Concerning the costs of adopting 3D printing as a new manufacturing technology to produce pharmaceutical formulations could include the costs of the initial investment and the annual maintenance costs of the printer, the personnel operating the printer, the raw materials, the packaging materials and the disposable manufacturing equipment.

### 5.4. Future Role of Pharmacy in 3D Pharmaceutical Formulation

Pharmacists are one of the first professions to recognize the true potential of 3D printing technology for medicine manufacturing. The pharmacist’s role will be to formulate and design customized formulations based on specific patients’ needs using the 3D printer. The pharmacy staff, as drug experts, can recommend the most suitable strategy and determine when the 3D printer might be applied in pharmaceutical applications. Clinical pharmacists working alongside university researchers, physicians, pharmaceutical companies and clinical trial units will successfully enhance this technology and the mindset shift in the pharmaceutical field [89].

## 6. Conclusions

This research clearly illustrates the feasibility of using extrusion-based 3DP techniques in the pharmaceutical industry and in hospital pharmacies. From a future perspective, the 3D printer will lead to important innovations for the future and bridge existing gaps in the supply of appropriate medical products for the pediatric population. The pharmaceutical applications of 3DP technology remain in the early stages, but many steps forward have been taken.

Nowadays, 3D printing is not widely used in pediatric pharmaceutical preparations. The main reasons are due to FDA regulations but also because of the lack of fixed guidelines on 3D-printed solid oral pharmaceutical dosage forms. But thanks to the efforts of researchers and sufficient feasibility data, a revolution is expected in 3DP.

In order to overcome the differences between the two legislatures, the FDA and the EMA developed a partnership in order to share information to avoid exposing children to unnecessary clinical trials. This pediatric collaboration aims to enhance science and reduce risks to children during pediatric drug development.

The extrusion-based process therefore appears to be a very promising technique for producing oral dosage forms for precision medicine aimed at the pediatric population. This will then make it possible to decentralize manufacturing in hospital pharmacies.

## Data Availability

The data presented in this study are available in this article.
